# Usefulness of antero-posterior radiograph and variability of management in non-major thoracolumbar injuries: a single centre pilot study and review of literature

**DOI:** 10.1186/s41016-018-0136-5

**Published:** 2018-10-15

**Authors:** Jason Yuen, Wisam Selbi, Lucy Lee, Tim Germon

**Affiliations:** 1 South West Neurosurgery Centre, Derriford Hospital, Plymouth, PL6 8DH UK; 2 Department of Radiology, Derriford Hospital, Plymouth, PL6 8DH UK

**Keywords:** (MeSH): spinal fractures, Spinal injuries, X-ray film

## Abstract

**Background:**

Most adult trauma protocols suggest that where there has been a dangerous mechanism of injury or the patient exhibits abnormal physiology, CT scan is the primary radiological investigation. Other patients who may have suffered thoraco-lumbar (T-L) trauma initially have antero-posterior (AP) and lateral plain X-rays performed. Our clinical experience suggests AP views are not particularly useful in the management of these relatively low-velocity injuries. This is the first study intended to determine the contribution made by AP X-rays in these cases.

**Methods:**

Adults with a history of T-L trauma referred to our tertiary spinal service over 20 weeks were reviewed. Those with a CT scan performed prior to X-rays were excluded. Four spine surgeons and four neuroradiologists were independently shown lateral X-rays along with the clinical details and asked to provide a management plan. Then they were shown the AP X-rays and asked if they would like to change their advice.

**Results:**

Fifty-two patients were identified. Thirty-four sets of supine and 40 sets of erect X-rays were included (four people only had lateral X-rays performed), yielding 1152 film views. Average patient age was 58.3 years with 30 (58%) males. Forty-five (87%) were AO type A (compression-type) fractures. Seven (13%) had been erroneously referred with a diagnosis of acute fracture, which on review was not considered to be the case. Fifty-four percent of fractures were between T11 and L2. Forty-six percent appeared osteoporotic.

In no instance did evaluation of the AP X-ray change the management plan which had been suggested following the evaluation of the lateral X-ray alone. However, there was significant variation in advice on further management between consultants.

**Conclusions:**

Our results suggest AP X-rays do not contribute to the management of low-velocity thoraco-lumbar traumas. Larger studies are required to support these findings, but there appears to be a potential to reduce both cost and radiation exposure. More importantly, it demonstrates there is large variability in the management of such patients due to the lack of evidence-based protocols.

## Background

### “Non-major” spinal trauma

Fracture management aims to prevent or reduce deformity and to promote healing. With Thoraco-lumbar (T-L) trauma, the use of clinical examination alone is a poor predictor for determining the need for imaging and intervention, unless the patient is asymptomatic, compos mentis *and* there is no significant mechanism of injury [1, 2]. Otherwise, the injury must first be identified and characterised using plain X-rays or computed tomography (CT) scans. Current evidence [1, 3-8] suggests those with the followings should have a low threshold to be imaged: signs such as focal tenderness or neurological deficit; those with a high-energy mechanism; presence of another spinal injury; painful distracting injury; depressed mental status; age over 60 years.

A literature review [9] concluded most patients with *major* blunt trauma require CT to screen for other injuries, e.g. visceral injuries, and it would also allow screening for bony spine injuries. However, the current evidence fails to clearly define the criteria used to decide the optimal imaging modality (plain radiographs vs CT scans). In particular, no study has conducted long-term follow-up on their trauma patients to identify all cases of spinal injury missed in the acute setting.

According to the National Institute for Health and Care Excellence (NICE) guidelines [10], patients who suffer suspected thoracic or lumbosacral spine injury with abnormal neurological signs or symptoms should have a CT scan first; otherwise, XR should be performed as a first-line investigation. They define major trauma as “an injury or combination of injuries that are life-threatening and could be life-changing because it may result in long-term disability” [11].

The trauma protocol at our institute endorses a similar approach. Where there has been a dangerous mechanism of injury (Table 1) or the patient exhibits abnormal physiology (Table 2), a CT scan is the primary radiological investigation.

**Table 1:** List of exampled “dangerous mechanism of injury” used in the trauma protocol of our Centre. It is usually warranted to perform CT scanning in these circumstances

Fall over 3 m	
Pedestrian or pedal cyclist hit by a motor vehicle	
RTC over 40mph, or ejection from a vehicle, or death to another occupant of the vehicle	
RTC with rollover, extensive damage to vehicle, or extrication time more than 20 min	

*RTC* road traffic collision

**Table 2:** List of exampled “abnormal physiology” (values for adults) used in the trauma protocol of our Centre. It is usually warranted to perform CT scanning in these circumstances

Pulse < 50 or > 120 beats per minute	
Respirations < 10 or > 30 per minute, or cyanosis	
Systolic Blood Pressure < 90 mmHg	
Head injury with GCS < 14	

*GCS* Glasgow Coma Scale

This leaves a subgroup of patients who may have suffered T-L spinal trauma in whom plain X-rays are performed as the first radiological investigation. X-rays are a fast and a relatively low-dose way to evaluate alignment, spacing, bones integrity, and soft tissues and are readily available in most hospitals [12]. As with all possible fractures, these are usually evaluated by images taken at two orthogonal planes, typically lateral and antero-posterior (AP) views [13]. AP can help to assess coronal plane alignment, loss of vertebral body height and transverse process fractures.

### Value of AP X-ray radiographs

A range of radiological signs such as widening of the interspinous and interlaminar distance, translation of more than 2 mm, kyphosis of more than 20°, dislocation, height loss of more than 50% and articular process fractures have been shown to be of value in establishing instability [14]. The majority of parameters in evaluating T-L fractures are measured in the sagittal plane [13, 15], and clinical experience suggests that AP view in minor trauma is not very useful in the acute management, and even less so at follow-up.

A widely used classification system that helps to guide treatment, thoracolumbar injury classification and severity score (TLICS) [16], involves three clinical characteristics: injury morphology, integrity of the posterior ligamentous complex and neurological status of the patient. Without a CT scan, in the context of non-major trauma, most of this information should be identifiable from physical examination and a lateral film. An AP XR is unlikely to add much further information.

Stable injuries with no neurological compromise tend to be treated conservatively, but kept under observation, because progressive post-traumatic deformity, or development of neurological deficits, may require surgical intervention [17].

The purpose of the study is to investigate if lateral plain film alone would be enough to make a comprehensive decision in the management of people who have potentially suffered T-L trauma but do not qualify for CT scanning on presentation.

## Methods

All adult patients with a history of T-L trauma and suspected abnormal XR referred to the spinal service over 20 weeks were retrospectively reviewed in the study, using the Neurosurgical Departmental referral database. Those with a CT scan performed prior to X-rays were excluded. If patients had both erect and supine X-rays on the same day, only the erect X-rays were included in the study, as we assumed that the latter was probably the films on which the referrals were based. If they were performed on different days, both the erect and supine X-rays were included.

Four consultant spine surgeons (three neurosurgical and one orthopaedic) and four consultant neuroradiologists in our tertiary service were independently shown the lateral XR alone, along with the history and examination findings recorded on the referral database (i.e. the information given to the consultant on the day of the referral).

**Fig. 1:**
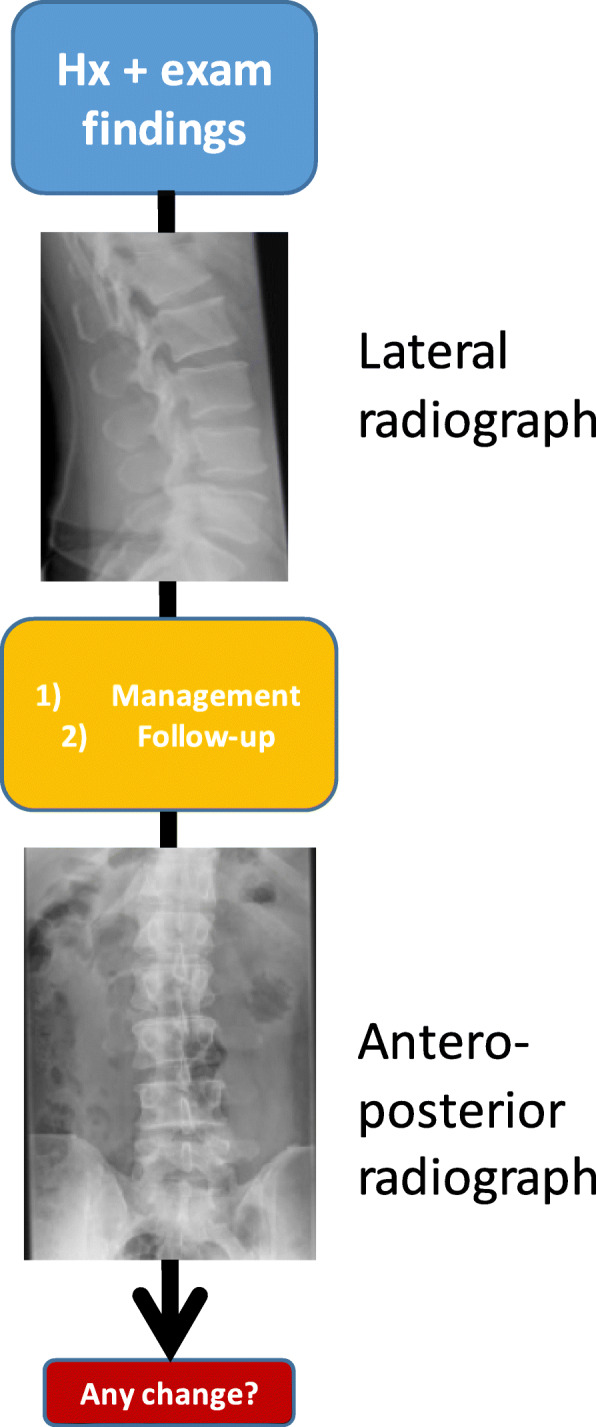
Schematic view of how data were collected from our subjects. Surgeon or radiologist is first shown the clinical details of the patient and then the lateral radiograph. They were asked to provide a management and/or follow-up imaging plan based on the XR. Afterwards, they were shown the antero-posterior radiograph and asked if they would like to change their advice. Any changes in the advice were recorded. *Hx* = history, see text for clarification

They were asked to provide a management and/or follow-up imaging plan based on the XR. Then, they were shown the AP XR and asked if they would like to change their advice. Any changes in the advice were recorded. A schematic view is shown in Fig. 1.

Management advice given by Consultants was classified into one of the following:

1. Surgeons
  - Review if pain persists
  - Erect XR
  - CT
  - MRI
  - 2-week follow-up
  - 6-week follow-up
  - Repeat XR after mobilisation
  - Others

2. Radiologists
  (i) CT
  (j) MRI
  (k) CT + MRI
  (l) XR of other parts
  (m) Erect XR
  (n) Repeat XR in 6 weeks
  (o) None

Statistical analysis was performed with SPSS 20.0 for Windows (SPSS Inc., Chicago, IL, USA). Cohen’s kappa tests were performed to compare differences in scores. Statistical significance was set at *p* < 0.05.

### Outcomes

The actual outcomes of patients were recorded from medical notes and clinic letters, in particular, whether or not they had any intervention and if they had been invited to the spinal fracture clinic.

## Results

**Fig. 2:**
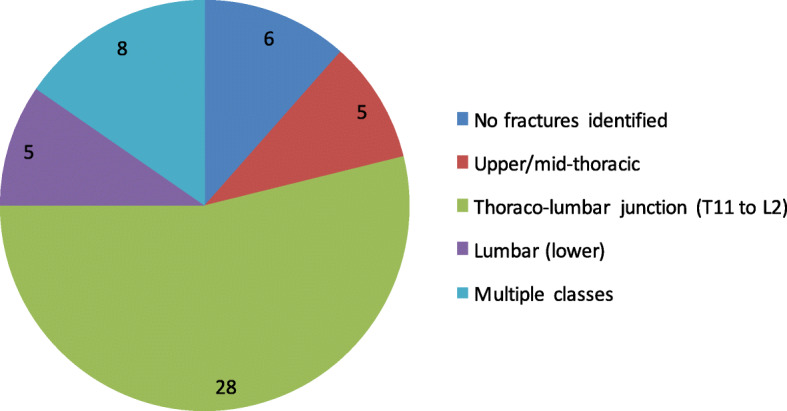
Distribution of anatomical location of fractures of the patients included in the study

In total, 52 patients were included. Mean age was 58.3 years (standard deviation, SD, 18.9 years). Thirty (58%) of the patients were male. They were all new referrals, and none had a neurological deficit. Using the AO classification [18], there were 45 type A fractures (not further subclassified; see the rationale in the “Discussion” section), and in 7 (13%), no acute fracture was unidentified. Twenty-four (46%) of the fractures appeared to be osteoporotic. The distribution of fracture locations is shown in Fig. 2. During the study period, there were 218 trauma CT scans done during the same period, although not all of which were necessarily done for suspected spinal trauma.

There were 34 supine and 40 erect films, of which 70 had AP and lateral films and four cases where only lateral erect XR was performed. As eight consultants were included in the study, 8 × [(70 × 2) + 4] = 1152 film examinations were included in this study. In the exceptions where only lateral films were performed, the subjects were asked hypothetically if an AP film would change the management plan.

*Not one case was identified where the presence of an AP film changed the management plan*. In the four cases without AP XR, all of the consultants (surgeons and radiologists) agreed that AP XR would unlikely to change the management.

### Intra-specialty variations in further management

**Fig. 3:**
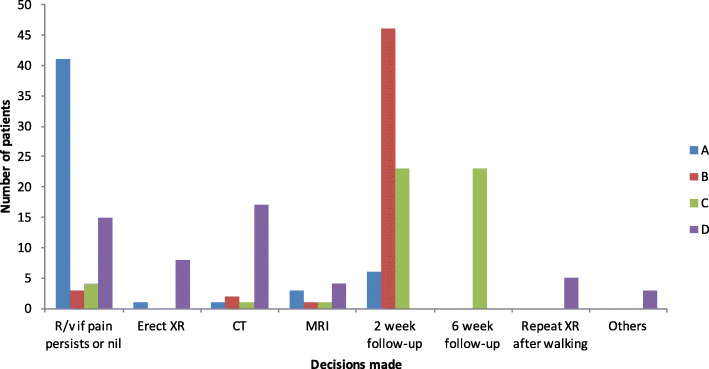
Follow-up management plan made by consultant spinal surgeons. (A) to (D) denote the individual surgeons. The vertical axis denotes the number of patients with a particular decision. The horizontal axis denotes the decisions advised

In no cases did all four *surgeons* agree on the same management plan. The distribution among surgeons is shown in Fig. 3. Table 3 denotes the kappa values between subjects.

**Table 3:** The *kappa* values of a head-to-head comparison between management plans made by consultant spinal surgeons. The standard errors are put in brackets. “A to D” denote the individual subjects. Please see the “Discussion” section for the interpretation of kappa values

	A	B	C	D
A	–	0.028 (0.031)	− 0.084 (0.038)	0.062 (0.047)
B	–	–	0.075 (0.049)	− 0.012 (0.017)
C	–	–	–	0.009 (0.021)
D	–	–	–	–

**Fig. 4:**
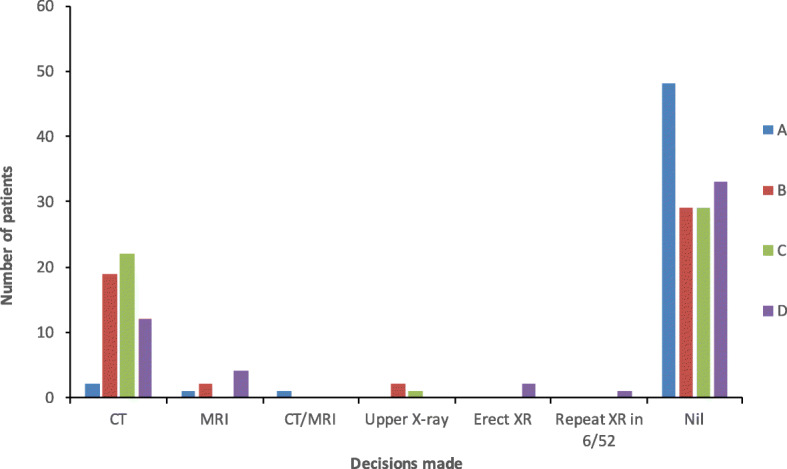
Follow-up imaging plan made by consultant neuroradiologist. (A) to (D) denote the individual radiologists. The vertical axis denotes the number of patients with a particular decision. The horizontal axis denotes the decisions advised

Among the *radiologists*, 19 patients’ plans were agreed by all four subjects. Their distribution is shown in Fig. 4. Table 4 denotes the kappa values between subjects.

**Table 4:** The *kappa* values of a head-to-head comparison between management plans made by consultant neuroradiologists. The standard errors are put in brackets. “A to D” denote the individual subjects. Please see the “Discussion” section for the interpretation of kappa values

	A	B	C	D
A	–	0.142 (0.087)	0.016 (0.063)	0.048 (0.077)
B	–	–	0.459 (0.109)	0.312 (0.105)
C	–	–	–	0.264 (0.102)
D	–	–	–	–

### Outcomes

**Fig. 5:**
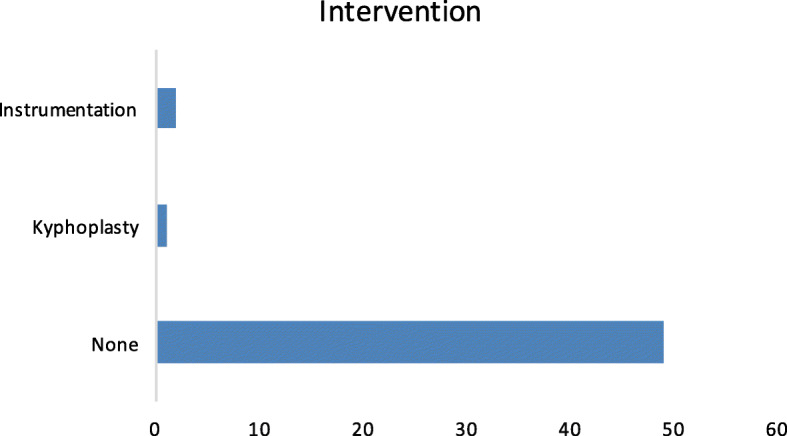
Outcome of patients in this study, in terms of surgical interventions provided

**Fig. 6:**
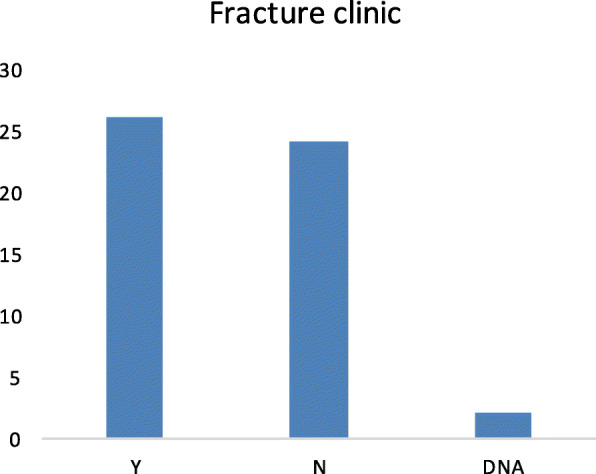
Number of patients who were offered to be followed up in clinic. *Y* denotes those who were invited to the clinic, and *N* denotes those who were not. *DNA*, do not attend

The outcomes are shown in Fig. 5. The majority had no active intervention. Figure 6 shows that more than half (54%) were invited to fracture clinics.

## Discussion

### AP radiographs

Over the past decades, the increased prevalence of CT scanners has dramatically changed the practice of spine imaging in the context of trauma. Evidence shows that they are more sensitive and accurate than plain films, especially in high-speed trauma [2, 19, 20]. A study even suggested that they have similar mean overall spinal imaging cost per patient, taking into account the cost of repeat imaging [21]. However, plain films remain a fast, safe and easily accessible modality for the imaging of patients with non-major T-L trauma, who are at very low risk of having suffered an unstable injury. Although it remains common practice to perform both AP and lateral XR (and our experience is that most radiographers would perform both unless instructed otherwise), there is actually little evidence that the AP is of additional value to the management. The results of our study support our hypothesis that the AP XR in this group does not contribute to subsequent management. To our knowledge, this is the first study to demonstrate that.

The majority of fractures in our study occurred at the thoraco-lumbar junction, consistent with the population described in the literature [22].

### Financial implications

According to the NICE guidelines, spinal X-rays (AP or lateral) cost £30 each [23]. The samples in our study had a total of 70 AP films between them, yielding an average cost of £40 per patient, which is of doubtful clinical value. This does not take into account the further X-rays on follow-up.

### Radiation implications

Radiation doses for AP and lateral lumbar films are 2.20 and 1.50 mSv, respectively (a typical chest XR results in a dose between 0.06 and 0.25 mSv) [24]. Each AP XR is thus equivalent to almost 36 chest X-rays. As patients may need repeated X-rays to access their injury, radiation exposure is a significant consideration. As a comparison, the dose of a CT scan of the spine is typically 6 mSv [25].

### Variation of practice

The sub-analysis of intra-specialty variation revealed that there is much difference in practice between consultants, even within the same department. As shown in the “Results” section, the kappa values (which measure inter-rater variability for qualitative outcomes) among surgeons vary between − 0.084 and 0.075, whereas those among radiologists vary between 0.048 and 0.459. Although the interpretation of kappa values has no universally accepted definition, one paper proposed the following as standards for strength of agreement: ≤ 0 = poor, 0.01–0.20 = slight, 0.21–0.40 = fair, 0.41–0.60 = moderate, 0.61–0.80 = substantial and 0.81–1.00 = almost perfect [26]. This variation almost certainly reflects the lack of data on the natural history and hence optimal management strategy for these low-velocity injuries. Therefore, we need formal prospective studies in order to resolve these uncertainties.

### Outcome study

Most of the patients were managed conservatively in this study, as we would expect from low-velocity type A fractures. Only approximately 50% were offered a clinic follow-up. This may be attributed to different spinal surgeons’ preference to use the service to follow patients up.

According to a recent literature review [27], there is a lack of standard of care for patients with vertebral compression fractures. Most patients with stable fractures tend to be treated by conservative treatment such as analgesia at the first instance but there are no clear guidelines defining at what point treatment such as surgery or vertebroplasty should be considered. No consistent radiological biomarkers have been developed to guide treatment strategies [27]. The NICE guidelines [28] recommend percutaneous vertebroplasty and percutaneous balloon kyphoplasty as options for treating osteoporotic vertebral compression fractures only in those who have severe ongoing pain after a recent, unhealed vertebral fracture despite optimal pain management and in whom the pain has been confirmed to be at the level of the fracture by physical examination and imaging. It concluded that “there were likely to be very few patients for whom these procedures were appropriate more than 12 weeks after fracture, and the appropriate timing in relation to the age of the fracture could be left for clinicians to judge.”

The American Academy of Orthopaedic Surgeons (AAOS) recommends against vertebroplasty for patients who present with an osteoporotic compression fracture on imaging and who are neurologically intact, but kyphoplasty is an option despite only limited evidence available [29].

Although there is a wealth of literature to help to determine between operative and non-operative options [30, 31], there is a lack of class 1 evidence for the timing of follow-up for these patients on which to base an informed decision.

### Limitations

The main limitation of our pilot study is the relatively small sample size, and we encourage bigger studies to support our findings. Also, this study contains a mixed group of erect and supine radiographs, which reduces the homogeneity of the samples.

It is difficult to perform *inter*-specialty comparisons, as the role of a surgeon is different from that of a radiologist. A radiologist would only advise on further imaging whereas a surgeon could advise on follow-up and interventions. However, both should have a common goal.

During our classification of fractures, we only used imaging X-rays to infer the type. According to the up-to-date AO classification [32], the exact typing requires detailed examination of ligaments as well, which often requires more sophisticated imaging such as CT and magnetic resonance imaging. Therefore, we have not been able to describe the exact typing of fractures, but this should not change the essential message of this paper.

Regarding the outcome data, we would encourage long-term data with a bigger patient cohort to be included in future studies.

## Conclusions

To the best of our knowledge, this is the first study to suggest that AP X-rays do not contribute to the formulation of management in low-velocity thoraco-lumbar injuries. There is potential to reduce both cost and radiation exposure to patients. We found a significant difference in management plans between consultants among spinal surgeons and among neuroradiologists. We encourage larger studies to be conducted to further inform optimal management strategies for conservatively managed low-velocity injuries.

## Abbreviations

AP: Antero-posterior
T-L: Thoraco-lumbar
XR: X-ray radiograph

## Authors’ information

JY and WS are neurosurgical registrars (residents) at Derriford Hospital (Plymouth)/Southmead Hospital (Bristol) in Southwest England, both with a special interest in trauma. LL is an experienced consultant neuroradiologist, and TG is the senior spinal neurosurgeon of the Centre.
